# DNA damage modulates interactions between microRNAs and the 26S proteasome

**DOI:** 10.18632/oncotarget.1957

**Published:** 2014-05-09

**Authors:** Anna S Tsimokha, Valentina A. Kulichkova, Elena V. Karpova, Julia J. Zaykova, Nikolai D Aksenov, Anastasia A. Vasilishina, Andrei V. Kropotov, Alexey Antonov, Nikolai A. Barlev

**Affiliations:** ^1^ Institute of Cytology, Russian Academy of Sciences, 194064 St. Petersburg, Russia; ^2^ Department of Biochemistry, University of Leicester, Leicester, LE1 9HN; ^3^ MRC Toxicology Unit, Leicester, LE1 9HN; ^4^ Molecular Pharmacology laboratory, Saint-Petersburg Institute of Technology, Saint-Petersburg 190013, Russia

**Keywords:** 26S proteasome, miRNA, DNA damage, doxorubicin

## Abstract

26S proteasomes are known as major non-lysosomal cellular machines for coordinated and specific destruction of ubiquitinylated proteins. The proteolytic activities of proteasomes are controlled by various post-translational modifications in response to environmental cues, including DNA damage. Besides proteolysis, proteasomes also associate with RNA hydrolysis and splicing. Here, we extend the functional diversity of proteasomes by showing that they also dynamically associate with microRNAs (miRNAs) both in the nucleus and cytoplasm of cells. Moreover, DNA damage induced by an anti-cancer drug, doxorubicin, alters the repertoire of proteasome-associated miRNAs, enriching the population of miRNAs that target cell cycle checkpoint regulators and DNA repair proteins. Collectively, these data uncover yet another potential mode of action for proteasomes in the cell via their dynamic association with microRNAs.

## INTRODUCTION

Proteasomes are multi-protein complexes, widely known to participate in protein degradation by ubiquitin-dependent and ubiquitin-independent proteolysis. The 26S proteasome consists of the 20S catalytic particle (CP) and the 19S regulator particle (RP). The 20S proteasome is a barrel-shaped structure formed of four heptameric rings: the two outer rings are made of alpha-type subunits and the two inner rings are made of beta-type subunits. Beta-subunits are responsible for proteolysis, while the main function of alpha-rings is to regulate an access of a substrate to the proteolytic chamber [1, 2].

The 19S regulatory particle is attached at either one or both ends of the CP and is essential for substrate recognition and preparation for destruction. Poly-ubiquitinylated substrates are recognised either by the integral ubiquitin receptors subunits, Rpn10 and Rpn13 [3] [4, 5], or by the proteasome-associated factors that also have affinity for ubiquitins (e.g. Rad23B)[6]. Subsequently, the substrate proteins undergo de-ubiquitinylation mediated by the metalloprotease Rpn11 prior to their degradation [7, 8]. The RP consists of 19 different subunits, including six regulatory AAA-ATPase subunits (Rpt1-6) and 13 regulatory non-ATPase subunits (Rpn1-3, Rpn5-13, and Rpn15). The AAA-ATPases form a hexameric ring that unfolds substrates using the energy of ATP. This step is important for efficient entering the amino-terminal end of the target protein into a narrow channel of the 20S CP.

Since efficient protein degradation is critical for cell cycle progression, proteasome inhibitors are considered to be potent anti-cancer drugs and several of them are currently in clinical trials [9]. Bortezomib (PS431) was the first proteasome inhibitor to be approved by FDA for the treatment of multiple myeloma [10]. This success made proteasomes an appealing therapeutic target and highlighted the importance of studying the regulatory mechanisms that control proteasome activities. Given the fact that genotoxic stress damages many proteins, which should then be utilized by proteasomes, it was perhaps not very surprising that the combinatorial treatment of multiple myeloma patients with bortezomib and genotoxic drugs (e.g. doxorubicin) resulted in synergistic effect [11].

Doxorubicin (DR) is an anti-cancer drug that belongs to the family of anthracycline inhibitors of topoisomerase II [12]. DR was reported to regulate the ubiquitin-proteasome system [13] and enhance the degradation of some transcription factors [14, 15]. Moreover, proteasomes were shown to interact with DR directly and carry it from cytoplasm into the nucleus [16]. A more detailed analysis of this phenomenon has revealed the critical role of the 20S CP subunits in binding doxorubicin [17].

In addition to “canonical” proteolytic activities proteasomes also possess several “non-canonical” activities [18]. In this respect, it is important to note that several decades ago a number of reports described the proteasome (at least the 20S CP) as an RNA binding protein complex [19, 20]. Moreover, the same group of authors was able to show that the CP is also associated with nuclear matrix [21, 22] as well as with the cytoskeleton [23]. Interestingly, these 20S CP, or prosomes, displayed RNase activities [24, 25]. Subsequent studies have identified the alpha5 subunit of CP as the one possessing RNAse activity. The latter can be regulated by various post-translational modifications in response to different environmental cues [18, 26]. Finally, the work from our group indicates that proteasomes may be involved in regulation of alternative splicing [27].

That proteasome preparations often contain small RNA species ranging from 20 to 120 nucleotides [28] prompted us to test whether proteasome-associated RNAs (pa-RNAs) belonged to the family microRNAs (miRNAs). Given the fact that DNA damage controls both proteolytic and endonuclease activities of proteasomes, we also examined how genotoxic stress induced by DR affected the repertoire of these small RNAs associated with the proteasome.

## RESULTS

### Doxorubicin elicits apoptosis in K562 cells

**Figure 1:**
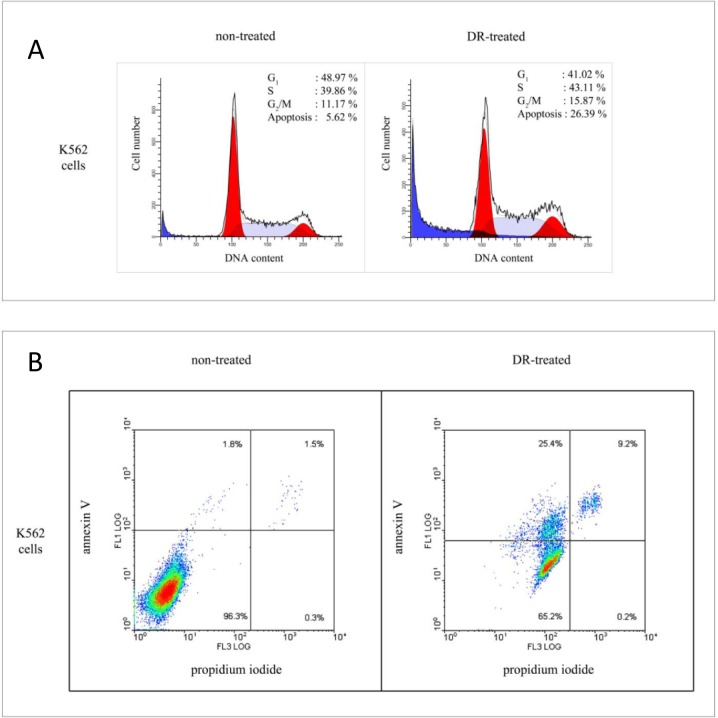
Cell cycle distribution and apoptotic response of K562 cells on doxorubicin treatment. (A) Non-synchronized K562 cells were non-treated (left) or treated (right) with 4 μM doxorubicin (DR) for 24 h before staining with propidium iodide for subsequent FACS analysis. Graphical presentation of the cell cycle data was obtained using Summit software. (B) Effect of doxorubicin on the ratio of apoptotic versus necrotic cells. Non-treated (left) and DR-treated (right) K562 cells were stained by annexin V (y axis) and propidium iodide (x axis). Cell population located in the lower left quadrant (negative staining for both Annexin V and propidium iodide) represent intact cells, cells permeable to propidium iodide located in the right quadrant represent necrotic and late apoptotic fraction, and the cells stained by Annexin V located in the upper left quadrant represent the apoptotic fraction. The numbers shown in each quadrant denote the percentage of intact, necrotic and apoptotic cells, respectively.

We and others have shown previously that proteasomes co-purify with a fraction of RNA species ranging from 1000 to 18-20 nucleotides [28, 29]. In fact, the abundance of the RNA fraction associated with proteasomes was calculated and varied from 0.0016% up tо 0.2% of total RNA depending on the organism and specific tissue used for experiments [30]. We wanted to test whether genotoxic stress, known to regulate both proteolytic and RNAse activities of proteasomes, also affected the binding of RNA to proteasomes. To address this, we first determined the conditions of genotoxic stress that caused apoptosis in K562 cells, a promyelocytic leukemia cell line with high proteasome content. To this end, we treated or non-treated cells with 4 uM of doxorubicin (DR) for 24 hours. As evident from the FACS analysis data (Fig. 1A), such treatment caused a significant fraction of cells to undergo apoptosis compared to the non-treated cells (26.4% versus 5.6%, respectively). Furthermore, annexin V staining of the same pair of samples showed higher intensity in the case of treated cells versus the non-treated ones (Fig. 1B). Thus, we concluded that the concentration of 4 uM of DR was sufficient to induce DNA damage-induced apoptosis in K562 cells. Therefore we used these experimental conditions of genotoxic stress for our future experiments.

### Purified proteasomes are associated with low molecular weight RNA species

**Figure 2:**
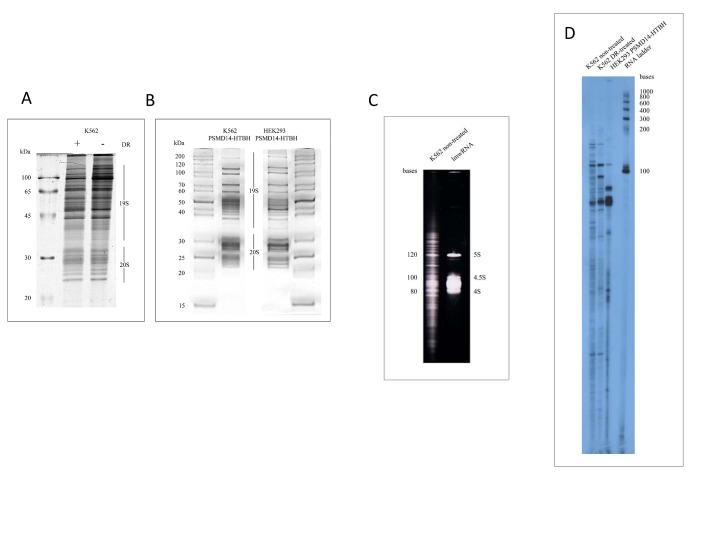
Analysis of small RNAs associated with purified proteasomes from different cell lines **(A)** Coomassie stains of 26S proteasomes purified from K562 cells non-treated or treated with doxorubicin. **(B)** Affinity-purified 26 proteasomes from K562 cells (left) and HEK293 (right) cells stably expressing PSMD14-6His-Tev-Biotin binding domain (HTB). Positions of 19S and 20S sub-complexes in the gel are shown. Markers of molecular weight (Fermentas Life Sciences) are indicated on the left **(A)** and on both sides in panel **(B)**. **(C)** Electrophoregram of the RNA component associated with proteasomes purified from K562 cells. Low molecular weight markers are shown on the right (5S, 4.5S and 4S RNA species correspond to 120 nt, 100nt, and 80 nt, respectively). **(D)** ^32^P-labeled lmw RNA species isolated from conventionally (K562 cells non-treated and treated with DR) and affinity purified proteasomes (HEK293 cells expressing PSMD14-HTB). Markers of molecular weight (Fermentas Life Sciences) are indicated on the right. RNA species were separated by electrophoresis in denaturing 8% PAAG with 8 M urea.

Next, we analysed the effect of DNA damage on the composition of 26S proteasomes (Fig. 2A). In agreement with the previously published data we did not detect gross changes in the composition of proteasomes purified from cells treated or non-treated with DR, although several subunits exhibited abnormal mobility in the gel due to post-translational modifications [26]. To visualise the spectra of RNA associated with proteasomes from non-treated and DR-treated cells the proteasome-associated RNA (pa-RNA) was extracted from the respective proteasome preparations and was subsequently end-labelled with [5'-P^32^] pCp in the presence of T4 RNA ligase (Fig. 2D). To exclude the possibility that pa-RNA was a contamination resulting from insufficient purity of samples we also extracted RNA from the highly affinity-purified preparation of proteasomes from both K562 and HEK293 cells (Fig. 2B). As can be judged from both non-labelled (Fig. 2C) and radioactively-labelled images (Fig. 2D) pa-RNA species ranged from 150 to 10-15 nucleotides (nt) in all three samples analysed. Moreover, despite all three samples contained common pa-RNA species of approximate size of 110 and 80 nt, they were quite different otherwise (Fig. 2D). The most pronounced differences between pa-RNAs from the DR-treated and non-treated cells were observed in the regions between 100 and 150 nt and 15 to 50 nt.

### Microarray analysis reveals a number of miRNAs associated with proteasomes in a cell compartment- and DR-specific manner

**Figure 3:**
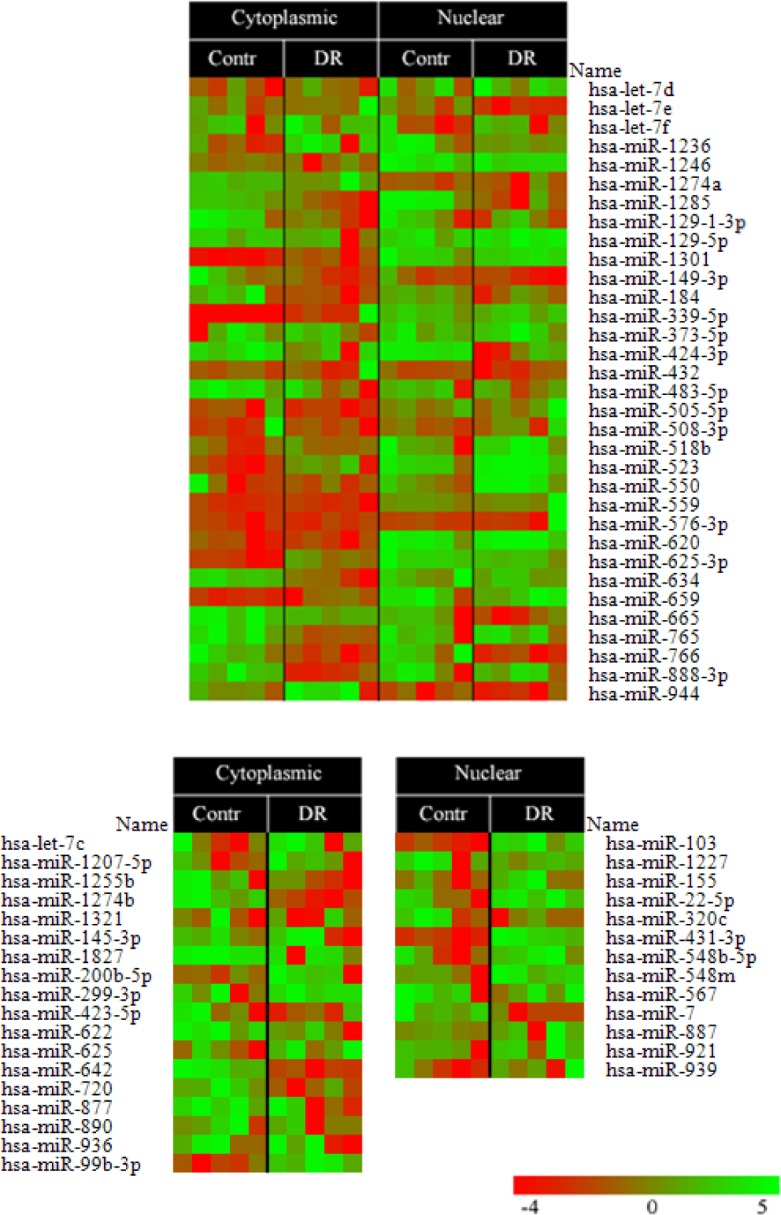
A heatmap of miRNA expression levels isolated from cytoplasmic and nuclear proteasomes non-treated (Contr) or treated with doxorubicin (DR) Two micrograms of proteasome-associated RNA (pa-RNA) were used for hybridization with custom microarrays of miRNAs. Fold difference in hybridization signal is shown as a colored bar where red color denotes the lowest signal and the green one – the highest.

The fact that pa-RNAs from DR-treated versus non-treated cells differed significantly in their low molecular weight species prompted us to explore whether these RNAs belonged to non-coding miRNAs. To address this question, we decided to employ microarray analysis using custom prepared arrays of micro-RNA available from miRBase v9.6 (http://www.mirbase.org/). Furthermore, if true, we wanted to compare the spectra of proteasome-associated miRNAs (pa-miRNAs) isolated from different cellular compartments (nucleus versus cytoplasm) of cells non-treated or treated with DR. Thus, we isolated pa-RNAs from cytoplasmic and nuclear fractions of K562 cells non-treated and treated with DR. Next, the resulting pa-RNAs were labelled and hybridized to the custom-made miRNA expression arrays [31] (Fig. 3). The results of microarray analysis confirmed the presence of miRNAs in the samples of purified proteasomes. Surprisingly, we detected a number of pa-miRNAs both in the nucleus and cytoplasm of both K562 and HEK293 cell lines (Fig. 3 and data not shown). Furthermore, DR treatment elicited specific changes in the levels of several pa-miRNAs purified from both nuclear and cytoplasmic fractions (e.g. *let-7, miR-103, miR-625, miR-634, and miR-944*).

### Validation of pa-miRNA presence in different cell types by (Q)RT-PCR

**Figure 4:**
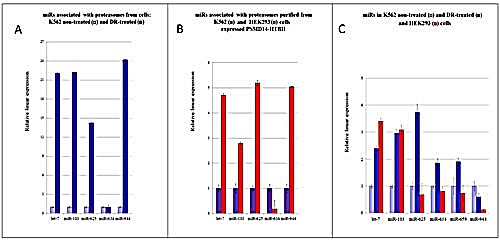
(Q)RT-PCR comparative analysis of pa-miRNAs levels in K562 and HEK293 cells **(A)** Comparison of expression levels of individual pa-miRNAs isolated from K562 cells non-treated or treated with DR. Changes in microRNA levels were determined by QRT–PCR and normalized against the U6 RNA signal. The values are presented as mean fold change ± SEM (n = 3). **(B)** Comparison of expression levels of individual pa-miRNAs from the DR-treated K562 and HEK293 cells. **(C)** Cellular levels of individual miRNAs in HEK293 cells and K562 cells non-treated or treated with DR. Samples were normalized against the *miR-659* signal, which was identical in these cells. Levels of microRNAs in non-treated K562 cells were arbitrary set as 1.

To confirm the validity of our microarray findings, we selected several pa-miRNA genes and validated their expression by QRT-PCR using specific primers (Fig. 4). Consistent with the microarray experimental data, expression levels of members of the let-7 family miRNAs were up-regulated in the pa-miRNA samples purified from K562 DR-treated cells compared to the ones purified from non-treated cells (Fig. 4A). In addition, *miR-103*, whose expression is known to be increased upon DNA damage, associated with nuclear proteasomes more avidly in cells treated with DR compared to the non-treated cells (Fig. 4A). Similarly, we detected higher contents of *miR-625* and *miR-944* in proteasomes purified from the DR-treated cells. On the contrary, the level of *pa-miR-634* slightly diminished upon DNA damage in K562 cells. It is important to note that several miRNAs exhibited differential association with cytoplasmic versus nuclear proteasomes irrespective of DNA damage (e.g. *miR-620, miR-339, miR-1301*). These results suggest that proteasomes, depending on the intracellular localisation, may be exposed to different pools of miRNAs. Alternatively, miRNAs are introduced to proteasomes by different accessory factors in cytoplasm compared to the nucleus.

To examine whether proteasomes bind specific miRNAs irrespective of the cellular context, we compared the expression levels of several pa-miRNAs isolated from proteasomes prepared from two different cell lines, K562 and HEK293, which represent suspension and adherent cells, respectively (Fig. 4 B). Most of pa-miRNAs tested were present in both preparations. Yet, it should be noted that the amounts of pa-miRNAs from HEK293 cells were consistently higher compared to the ones from K562. One exception was *miR-634*, which was significantly lower in the pa-miRNA fraction from HEK293 cells compared to the one from K562 cells.

We assumed that these variations may be due the difference in expression levels of specific miRNAs in these cell lines. If one assumes that miRNAs bind proteasomes non-specifically, then there should be a direct correlation between the intracellular levels of such miRNAs and their presence in the proteasome fraction. To test this hypothesis, we compared cellular levels of miRNAs *let-7*, *miR-103*, *miR-625*, *miR-634*, *miR-659* and *miR-944* in HEK293 and K562 cells treated or non-treated with DR and correlated their amounts with their relative content in the pa-miRNA fractions (Fig. 4C). Importantly, we observed no obvious correlation between the cellular levels of particular miRNAs and their amounts associated with proteasomes (compare Fig. 4B and 4C). For example, cellular levels of *miR-625* and *miR-944* in HEK293 cells are lower than in K562 cells but were 5-fold more abundant in the pa-miRNA fraction compared to the matching sample from K562 cells. Collectively, these results confirmed our notion that proteasomes specifically interact with a defined pool of miRNAs irrespective of their cellular abundance.

### The bioinformatics analysis of pa-miRNAs targets reveals preference for several DNA Damage Response pathways

**Figure 5:**
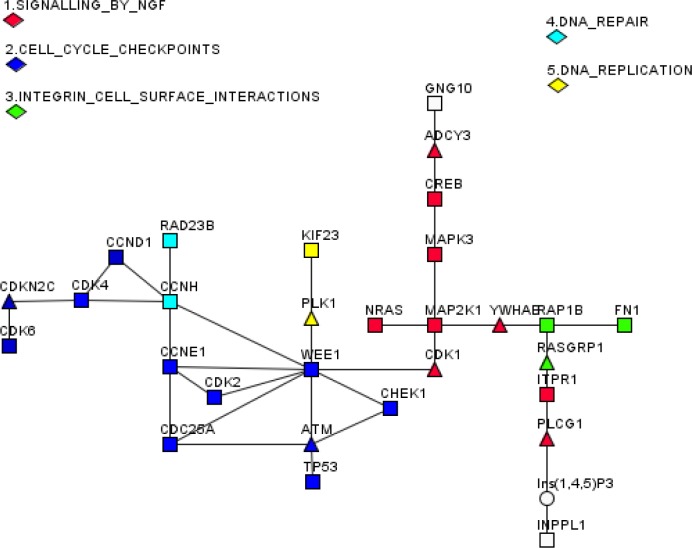
A connectivity map of genes targeted by DR-affected pa-miRNAs Genes that are targeted by pa-miRNA (squares), whose association with proteasomes is affected by DR treatment, were connected into five statistically significant cellular pathways with maximal gap of two intermediates (triangles). Connecting metabolites are shown as circles. Only those pa-miRNAs were scored as affected by DR and thus used for subsequent analysis, which showed >1.5 fold difference in the level of expression upon DR on at least three different probes.

Finally, we wanted to find out whether there is a specific pathway(s) that might be targeted by DR-regulated pa-miRNAs. To this end, we employed a bioinformatics approach. First, using experimentally verified targets of pa-miRNAs retrieved with MIRTARBASE software (http://mirtarbase.mbc.nctu.edu.tw) we generated a list of potential gene targets [32] for these miRNAs (Table 2). Next, we used the algorithm called R-spider [33, 34], which is based on the statistical framework that analyses united Reactome signalling and KEGG metabolic networks for gene- and gene-metabolite interactions. The aim of this statistical analysis was to connect maximal number of genes derived from Table 2 into statistically significant gene networks with maximum of two missing genes in each pathway (Fig. 5). Nineteen genes targeted by pa-miRNAs affected by DR could be connected into five networks with statistical confidence better than 0.05 based on the Monte-Carlo simulation of random gene entries (Fig. 5). Notably, the “cell cycle checkpoints” gene network including CHEK1, WEE1, TP53, CDK2, CCNE1, CDC25A, CCND1, CDK4, and CDK6 was enriched with much higher (p<0.01) statistical confidence compared to other networks. These results suggest that the pa-miRNAs fraction is enriched with miRNAs that target cell cycle regulators. Other gene networks include “signalling by NGF”, “integrin cell surface interactions”, “DNA repair” and “DNA replication”. All of these gene networks are affected by DNA damage suggesting that miRNA associated with proteasomes may be a part of global DNA Damage Response (DDR).

**Table 1:** A list of pa-miRNAs whose expression is altered by DNA damage and their target genes

Name of miR	Target Genes
miR103	CCNE1, GPD1, CDK2, DICER1, CREB
let7f	PRDM1, KLK10, KLK6
let7c	HMGA2, NRAS, TGFBR1, BCL2L1, DICER1
let7e	HMGA2 WNT1 EIF3J SMC1A
miR200B	EP300 WASF3 FN1 GATA4 PTPN12 ZEB1 SIP1 BAP1 ZFPM2 ETS1 MATR3 ZEB2 RERE
miR625	NTRK3
miR659	GRN
miR99b	RAVER2
miR1285	TP53
miR184	NFATC2, AKT2, INPPL1
miR424	CDC25A PLAG1 NFIA MAP2K1 KIF23 CHEK1 ANLN, CCNF CCNE1 FGFR1 CCND1 PIAS1 WEE1 HIF1A SIAH1 ITPR1 CUL2 CCND3 ATF6 SPI1 CDC14A CDK6
miR642	DOHH, LGMN, MDN1
miR765	NTRK3, HNF4A
miR766	HNF4A, XRCC6, TMEM120B, NUP205, WDR45B, ZNF48, CDK4, KAT5
miR483-5p	SRF, MAPK3, BBC3
miR518B	RAP1B
miR1301	MRPL10, ZNF816, GNG10, CCNH, RAD23B, HIST2H2AC, PARL, TXN, CREBBP, HMGA1
miR1236	ALS2CR12, KPNB1, H3F3B, SRSF5, KAT6B, PSMD11, FOXC1, XPO1
miR550	WNK1, COX1, DNAJA3, TXLNG
miR939	IL6, SH3BP2, AMPD2

**Table 2:** miRNA primer sequences used for verification of miRNA microarray results by QRT-PCR

Primer	Sequences (5' to 3')	Accession No. or reference article	Position
let-7e	TGAGGTAGGAGGTTGTATAGTT	MIMAT0000066	8-29
miR-103	AGCAGCATTGTACAGGGCTATGA	MIMAT0000101	48-70
miR-625	AGGGGGAAAGTTCTATAGTCC	MIMAT0003294	15-35
miR-634	AACCAGCACCCCAACTTTGGAC	MIMAT0003304	61-82
miR-659	CTTGGTTCAGGGAGGGTCCCCA	MIMAT0003337	61-82
miR-944	AAATTATTGTACATCGGATGAG	MIMAT0004987	54-75
U6	GGCAGCACATATACTAAAATTGGAA	[59]	

Specifically, NGF signalling is known to protect neuronal and hematopoetic cells from cell death [35]. By the same token, integrin cell surface interactions network is connected to DNA damage-induced apoptosis. Integrins regulate diverse pathways including activation of protein tyrosine and serine/threonine kinases, lipid kinases, and small GTPases [36]. It has been shown that in some cell types that survive in suspension, detachment from the Extracellular Matrix (ECM) decreases cell death in response to DNA damage. The mechanism involves integrin-dependent changes in the Arf protein and correlates with genetic instability [37].

Another pathway (DNA repair) involves Rad23B and CCNH target genes. In addition to its role in DNA repair, Rad23B serves as an adapter module between the poly-ubiquitinylated substrate proteins and proteasomes [38, 39]. The “DNA replication” network is represented by two genes: KIF23, a kinesin family member, and PLK1 kinase are both targets of DNA damage mitotic checkpoints, which also participate in mitosis [40]. In line with this, most of the pa-miRNA target genes belong to the group of cell cycle checkpoints genes (Fig. 5). Importantly, the target genes are involved in both G1/S (CCNE1, CDK2, CCND1, CDK4, CDK6, and CDKN2C (p18/INK4c)) and G2/M (CDC25A, Wee1) control. It should be noted that Chek1 regulates both intra-S and G2/M checkpoints [41, 42]. Thus, it can be concluded that the majority of pa-miRNA's target genes are involved in the regulation of cell cycle checkpoints and cell cycle progression.

## DISCUSSION

That proteasomal 20S particles were associated with low molecular weight (lmw) RNA species has been known for decades [19, 20, 28, 30]. However, neither the exact composition nor the function(s) of these lmw RNAs were characterised in details. In this report we demonstrate for the first time that at least some portion of these lmw RNAs belong to miRNAs and that genotoxic stress regulates the association of miRNAs with proteasomes.

Since the proteasome is a large multi-subunit complex with several enzymatical activities, including the endoribonuclease one (for review see [1, 2]), it is therefore possible that the association between miRNAs and proteasomes might be non-specific. However, if it is non-specific, then the profile of miRNAs associated with the proteasome fraction should mirror the profile of most abundantly expressed miRNAs in the cell. However, this was not the case, as no appreciable correlation was observed between the intracellular levels of miRNAs and their levels found associated with proteasomes (Fig. 4C). While comparing pa-miRNAs from different cell lines we noticed a core set of miRNAs that bound proteasomes irrespective of the cellular context, which further argues against the non-specific binding of miRNAs to proteasomes.

What is the molecular basis for interactions between miRNAs and proteasomes? It is plausible that miRNAs bind specific subunits of the 26S complex directly. In fact, our *in vitro* data indicate that certain individual alpha type subunits of the 20S complex can interact with miRNAs. However, we do not favour this scenario, given that the 20S complex has a strong net negative charge which should repulse also negatively charged molecules of miRNAs. Instead, we hypothesize that miRNAs are specifically targeted to proteasomes by miRNA binding proteins either directly or via yet additional accessory factors. One possibility is that these interactions might be mediated via the heat shock chaperon proteins. For example, both RISC miRNA processing complex and 26S proteasome require Hsp90 chaperon for their assembly and maintenance [43, 44]. It has been shown that Hsp90 stabilized Argonautes proteins of the RISC complex before binding RNA thus facilitating efficient loading of small RNA [43]. Moreover, the Hsp90 protein binds directly to the N-terminus of overexpressed mammalian Ago2 and stabilizes Dicer interactions with Ago2 [45, 46]. On the other hand, Hsp90 plays an important role in the assembly and maintenance of the 26S proteasome [47]. This protein is also instrumental for transporting the proteasome from cytoplasm to the nucleus [48]. Using a cell-free nuclear reconstitution system, Savulescu et al (2011) [48] found that the 20S CP species that was actively imported through nuclear pore complexes (NPCs) to the nucleoplasm contained Hsp90 compared to other CP species that could not be delivered to the nucleoplasm. These data are consistent with the data published earlier that proteasomes are highly enriched with Hsp90 proteins [49]. Our own observations that the alpha7 (PSMA3) subunit of the 20S complex interacts with Hsp90 are in line with this hypothesis [27]. Taking these facts together, it is likely that 26S proteasomes interact via Hsp90 with RISC complexes loaded with miRNAs and hence gain access to the pool of different miRNAs.

However, in addition to the Hsp90/RISC axis, other proteins may mediate interactions between miRNAs and proteasomes. For example, several transcription factors that include p53 and SMAD family members were shown to bind the miRNA processing complex, Drosha [50] [51, 52]. On the other hand, these transcription factors undergo ubiquitylation and hence may interact with proteasomes [53, 54]. Therefore, it is plausible that they can also mediate indirect recruitment of miRNA to proteasomes. Future studies should address this important question.

**Figure 6:**
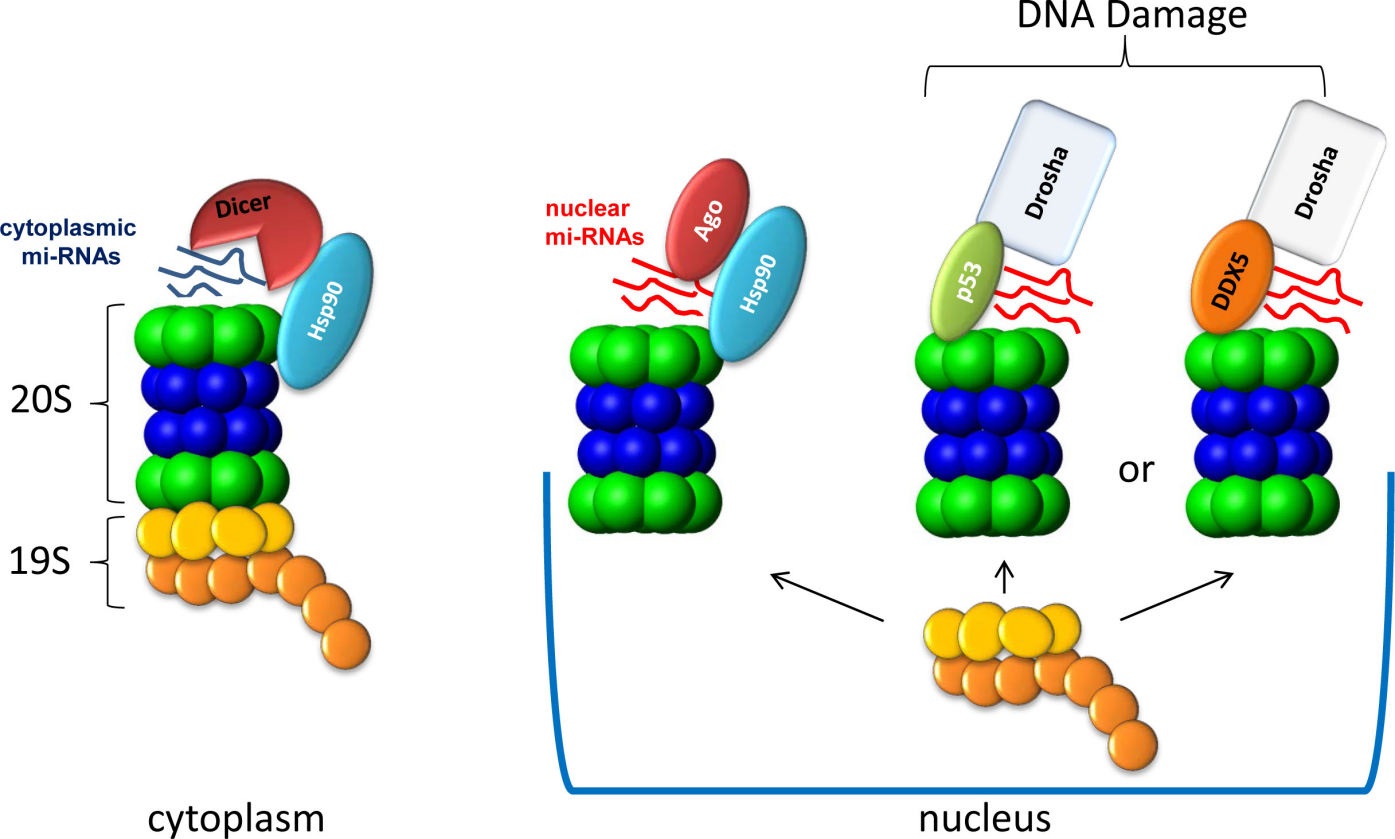
A model of possible interaction mechanisms between 26S proteasomes and miRNAs In cytoplasm, proteasomes may interact with miRNA via HSP90, which, in turn, binds the miRNA-processing complex, Dicer. In the nucleus, proteasomes may associate with miRNAs via similar mechanism (left), or through interactions with other proteins (e.g. p53 or DDX5), thereby recruiting additional miRNA-processing complexes (Drosha). The composition of proteasome bound with miRNAs in the nucleus is not clear.

Finally, our results unequivocally suggest that the DR-induced genotoxic stress affects the composition of miRNAs in both cytoplasmic and nuclear fractions of proteasomes. It is conceivable that genotoxic stress may affect the interactions of proteasomes with Hsp90 and/or specific miRNA-binding proteins (Fig. 6). Alternatively, the difference in pa-miRNA assortment conferred by genotoxic stress can be attributed to the cell cycle-restricted expression of certain miRNAs. Accordingly, DR treatment of K562 cells phase may facilitate the over-representation of G2/M-specific miRNAs in the pa-miRNA fraction (Fig. 1).

Importantly, our bioinformatics data indicate that pa-microRNAs can be allocated to several gene networks. Noteworthy, all of these networks are connected to DNA damage response, which points to a potential biological role of pa-miRNAs during genotoxic stress (Fig. 6). Future studies should address this important question.

## MATERIALS AND METHODS

### Cell cultures and treatment conditions

The human erythromyeloblastoid leukemia cell line K562, human embryonic kidney cell line HEK293 were purchased from the Russian Cell Culture Collection (Institute of Cytology, St. Petersburg, Russia). K562 cells were cultured in RPMI 1640 medium supplemented with 10% fetal bovine serum (FBS) and gentamycin. HEK293 cells and ecotropic Phoenix packaging cell line were grown in Dulbecco modified Eagle medium (DMEM), containing 10% FBS and penicillin/streptomycin 50 U/ml. HEK293 cells with stable expression of Rpn11-HTBH were generated by retroviral transfection with pQCXIP-PSMD14-HTBH vector (kindly provided by Dr. L.Huang) as described previously [55]. To induce DNA damage, the cells were incubated with 4μM doxorubicin (DR) for 24 hours.

### Cell cycle and apoptosis assay

To study cell cycle distribution, cells were harvested, washed with the PBS and permeabilized for 30 min with 0.01% saponin. Cells were washed with PBS and incubated with 0.1 mg/ml RNase A and 50 mg/ml propidium iodide (PI) for 15 min at 37 °C prior to analysis with an ATC300 cytometer (Brucker).

To determine the extent of apoptosis, cells were harvested, washed with the PBS and stained with FITC-conjugated annexin V and PI using the Annexin V kit (Santa Cruz) as recommended by the manufacturer. Thereafter, samples were analyzed by flow cytometry (Brucker) for the presence of viable (annexin V- and PI-negative), early apoptotic (annexin V-positive, PI-negative), and late apoptotic (annexin V- and PI-positive) cells. All tests were performed in duplicate.

### Isolation and purification of proteasomes

Non-treated and DR-treated K562 cells were lysed for 40 min at 4 °C in a buffer containing 50 mM Trise-HCl, pH 8.0, 150 mM NaCl, 0.1% SDS, 1x protease inhibitor cocktail (Roche). Proteasomes were extracted and purified from cytosol using a multistep purification procedure as described previously [56].

HEK293 cells with stable expression of Rpn11-HTBH were lysed for 30 min at 4 °C in 50 mM Trise-HCl, pH 7.5, 100 mM NaCl, 10% glycerol, 5 mM ATP, 1 mM DTT, 5 mM MgCl_2_, 1x protease inhibitor cocktail (Roche), and 0.5% NP-40. Cell lysates were incubated with high-capacity streptavidin agarose beads (Thermo Scientific) overnight at 4°C. Streptavidin-immobilized proteasomes were purified as described previously[55]. For each purification batch, 10 μg of purified proteasomes were analyzed for purity on 12% SDS-PAG.

### RNA manipulations

Total RNA from cultured cells was extracted using TRIzol reagent (Invitrogen) according to the manufacturer's instructions. The proteasome-associated RNAs (pa-RNAs) were extracted from the purified proteasome sample as follows: treatment with pronase (300μg/ml) and proteinase K (250μg/ml) in 10 mM Tris-HCl (pH 7.5) in the presence of 0.2% SDS at 37°C for 3 h. Next, psRNA was then isolated by phenol-chloroform extraction and ethanol precipitation. The pa-RNA samples were 3' end-labelled overnight at 4°C using [5'-P^32^]pCp (cytidine-3',5'-bis- phosphate) and T4 RNA ligase in 50 mM Tris-HCl, pH 7.9, 10 mM MgCl_2_, 3.3 mM DTT, and 10% DMSO. The resulting pa-RNAs were separated on a 8% PAG containing 8 M urea.

For microarray analysis, pa-RNAs were labelled and hybridized to miRNA microarray chips[31] as previously described[41]. Briefly, 2 μg of pa-RNA from each sample was biotin labelled during reverse transcription using random hexamers. Raw data were normalized and analysed by GeneSpring GX software version 7.3 (Agilent Technologies, Santa Clara, CA, USA) as described [57]. All microarray data were submitted as heatmaps generated by using Stanford software [58].

Real-time reverse transcription PCR quantification (QRT-PCR) of miRNAs were performed using 1 μg of total RNA or 0.4 μg of pa-RNA using the miScript Reverse Transcription Kit (Qiagen) and the miScript SYBR Green PCR Kit (Qiagen) according to the manufacturer's instructions. Complementary primer sequences for the mature forms of selected miRNAs were designed based on miRBase sequence data-base. The miRNAs analyzed included *let-7*, *miR-103*, *miR-625*, *miR-634*, *miR-659*, and *miR-944*. The primers used are presented in Table 2. Real-Time quantitative (Q) PCR was performed using ABI 7500 instrument (Applied Biosystems). Normalization was performed with U6 snRNA and *mir-659* for the total RNA and pa-miRNA, respectively. All reactions were performed in triplicates. Relative quantification was performed using the comparative cycle threshold (2^−ΔΔCt^) method.

### Bioinformatics

The list of miRNA differentially associated with proteasomes upon DR treatment was generated using threshold of >1.5. To generate the list of target genes for pa-miRNAs MIRTARBASE software (http://mirtarbase.mbc.nctu.edu.tw) was used. The R-spider algorithm implementation has been described previously [33, 34]. Statistically significant gene networks were visualized by Medusa software (http://graph-medusa.sourceforge.net/).

## Abbreviations

FITC: fluorescein isothiocyanate
DTT: dithiothreitol
DMSO: dimethyl sulfoxide
PI: propidium iodide
PAG: polyacrylamide gel
DR: doxorubicin

## References

[1] Mittenberg AG, Moiseeva TN, Barlev NA (2008). Role of proteasomes in transcription and their regulation by covalent modifications. Front Biosci.

[2] Konstantinova IM, Tsimokha AS, Mittenberg AG (2008). Role of proteasomes in cellular regulation. International review of cell and molecular biology.

[3] Lasker K, Forster F, Bohn S, Walzthoeni T, Villa E, Unverdorben P, Beck F, Aebersold R, Sali A, Baumeister W (2012). Molecular architecture of the 26S proteasome holocomplex determined by an integrative approach. Proc Natl Acad Sci U S A.

[4] Schreiner P, Chen X, Husnjak K, Randles L, Zhang N, Elsasser S, Finley D, Dikic I, Walters KJ, Groll M (2008). Ubiquitin docking at the proteasome through a novel pleckstrin-homology domain interaction. Nature.

[5] Husnjak K, Elsasser S, Zhang N, Chen X, Randles L, Shi Y, Hofmann K, Walters KJ, Finley D, Dikic I (2008). Proteasome subunit Rpn13 is a novel ubiquitin receptor. Nature.

[6] Chen L, Madura K (2002). Rad23 promotes the targeting of proteolytic substrates to the proteasome. Mol Cell Biol.

[7] Verma R, Aravind L, Oania R, McDonald WH, Yates JR, Koonin EV, Deshaies RJ (2002). Role of Rpn11 metalloprotease in deubiquitination and degradation by the 26S proteasome. Science.

[8] Yao T, Cohen RE (2002). A cryptic protease couples deubiquitination and degradation by the proteasome. Nature.

[9] Rastogi N, Mishra DP (2012). Therapeutic targeting of cancer cell cycle using proteasome inhibitors. Cell division.

[10] Adams J (2004). The proteasome: a suitable antineoplastic target. Nat Rev Cancer.

[11] Du BY, Song W, Bai L, Shen Y, Miao SY, Wang LF (2012). Synergistic effects of combination treatment with bortezomib and doxorubicin in human neuroblastoma cell lines. Chemotherapy.

[12] Tewey KM, Rowe TC, Yang L, Halligan BD, Liu LF (1984). Adriamycin-induced DNA damage mediated by mammalian DNA topoisomerase II. Science.

[13] Liu J, Zheng H, Tang M, Ryu YC, Wang X (2008). A therapeutic dose of doxorubicin activates ubiquitin-proteasome system-mediated proteolysis by acting on both the ubiquitination apparatus and proteasome. American journal of physiology Heart and circulatory physiology.

[14] Ito T, Fujio Y, Takahashi K, Azuma J (2007). Degradation of NFAT5, a transcriptional regulator of osmotic stress-related genes, is a critical event for doxorubicin-induced cytotoxicity in cardiac myocytes. J Biol Chem.

[15] Poizat C, Sartorelli V, Chung G, Kloner RA, Kedes L (2000). Proteasome-mediated degradation of the coactivator p300 impairs cardiac transcription. Mol Cell Biol.

[16] Kiyomiya K, Kurebe M, Nakagawa H, Matsuo S (2002). The role of the proteasome in apoptosis induced by anthracycline anticancer agents. Int J Oncol.

[17] Kiyomiya K, Matsuo S, Kurebe M (2001). Mechanism of specific nuclear transport of adriamycin: the mode of nuclear translocation of adriamycin-proteasome complex. Cancer Res.

[18] Kulichkova VA, Tsimokha AS, Fedorova OA, Moiseeva TN, Bottril A, Lezina L, Gauze LN, Konstantinova IM, Mittenberg AG, Barlev NA (2010). 26S proteasome exhibits endoribonuclease activity controlled by extra-cellular stimuli. Cell Cycle.

[19] Schmid HP, Akhayat O, Martins De Sa C, Puvion F, Koehler K, Scherrer K (1984). The prosome: an ubiquitous morphologically distinct RNP particle associated with repressed mRNPs and containing specific ScRNA and a characteristic set of proteins. EMBO J.

[20] Grossi de Sa MF, Martins de Sa C, Harper F, Olink-Coux M, Huesca M, Scherrer K (1988). The association of prosomes with some of the intermediate filament networks of the animal cell. J Cell Biol.

[21] De Conto F, Pilotti E, Razin SV, Ferraglia F, Geraud G, Arcangeletti C, Scherrer K (2000). In mouse myoblasts nuclear prosomes are associated with the nuclear matrix and accumulate preferentially in the perinucleolar areas. J Cell Sci.

[22] Ioudinkova E, Razin SV, Borunova V, de Conto F, Rynditch A, Scherrer K (2005). RNA-dependent nuclear matrix contains a 33 kb globin full domain transcript as well as prosomes but no 26S proteasomes. J Cell Biochem.

[23] Arcangeletti C, De Conto F, Sutterlin R, Pinardi F, Missorini S, Geraud G, Aebi U, Chezzi C, Scherrer K (2000). Specific types of prosomes distribute differentially between intermediate and actin filaments in epithelial, fibroblastic and muscle cells. European journal of cell biology.

[24] Jarrousse AS, Petit F, Kreutzer-Schmid C, Gaedigk R, Schmid HP (1999). Possible involvement of proteasomes (prosomes) in AUUUA-mediated mRNA decay. J Biol Chem.

[25] Jorgensen L, Hendil KB (1999). Proteasome subunit zeta, a putative ribonuclease, is also found as a free monomer. Mol Biol Rep.

[26] Moiseeva TN, Bottrill A, Melino G, Barlev NA (2013). DNA damage-induced ubiquitylation of proteasome controls its proteolytic activity. Oncotarget.

[27] Fedorova OA, Moiseeva TN, Nikiforov AA, Tsimokha AS, Livinskaya VA, Hodson M, Bottrill A, Evteeva IN, Ermolayeva JB, Kuznetzova IM, Turoverov KK, Eperon I, Barlev NA (2011). Proteomic analysis of the 20S proteasome (PSMA3)-interacting proteins reveals a functional link between the proteasome and mRNA metabolism. Biochem Biophys Res Commun.

[28] Schmid HP, Pouch MN, Petit F, Dadet MH, Badaoui S, Boissonnet G, Buri J, Norris V, Briand Y (1995). Relationships between proteasomes and RNA. Mol Biol Rep.

[29] Konstantinova IM, Kulichkova VA, Petukhova OA, Kozhukharova IV, Turoverova LV, Volkova IV, Il'kaeva OR, Ermolaeva Iu B, Teslenko LV, Mittenberg AG, Ignatova LN, Gauze LN (1996). [A novel class of small RNP (alpha-RNP) in human cell line K-562 and coordinated control of expression of Alu-containing messenger RNA]. Doklady Akademii nauk / [Rossiiskaia akademii nauk].

[30] Petit F, Jarrousse AS, Boissonnet G, Dadet MH, Buri J, Briand Y, Schmid HP (1997). Proteasome (prosome) associated endonuclease activity. Mol Biol Rep.

[31] Ferland-McCollough D, Fernandez-Twinn DS, Cannell IG, David H, Warner M, Vaag AA, Bork-Jensen J, Brons C, Gant TW, Willis AE, Siddle K, Bushell M, Ozanne SE (2012). Programming of adipose tissue miR-483-3p and GDF-3 expression by maternal diet in type 2 diabetes. Cell Death Differ.

[32] Antonov AV, Knight RA, Melino G, Barlev NA, Tsvetkov PO (2013). MIRUMIR: an online tool to test microRNAs as biomarkers to predict survival in cancer using multiple clinical data sets. Cell death and differentiation.

[33] Antonov AV (2011). BioProfiling. de: analytical web portal for high-throughput cell biology. Nucleic Acids Research.

[34] Antonov AV, Schmidt EE, Dietmann S, Krestyaninova M, Hermjakob H (2010). R spider: a network-based analysis of gene lists by combining signaling and metabolic pathways from Reactome and KEGG databases. Nucleic Acids Res.

[35] Soilu-Hanninen M, Ekert P, Bucci T, Syroid D, Bartlett PF, Kilpatrick TJ (1999). Nerve growth factor signaling through p75 induces apoptosis in Schwann cells via a Bcl-2-independent pathway. The Journal of neuroscience : the official journal of the Society for Neuroscience.

[36] Schwartz MA, Schaller MD, Ginsberg MH (1995). Integrins: emerging paradigms of signal transduction. Annual review of cell and developmental biology.

[37] Lewis JM, Truong TN, Schwartz MA (2002). Integrins regulate the apoptotic response to DNA damage through modulation of p53. Proc Natl Acad Sci U S A.

[38] Dantuma NP, Heinen C, Hoogstraten D (2009). The ubiquitin receptor Rad23: at the crossroads of nucleotide excision repair and proteasomal degradation. DNA repair.

[39] Brignone C, Bradley KE, Kisselev AF, Grossman SR (2004). A post-ubiquitination role for MDM2 and hHR23A in the p53 degradation pathway. Oncogene.

[40] Hood EA, Kettenbach AN, Gerber SA, Compton DA (2012). Plk1 regulates the kinesin-13 protein Kif2b to promote faithful chromosome segregation. Mol Biol Cell.

[41] Lezina L, Purmessur N, Antonov AV, Ivanova T, Karpova E, Krishan K, Ivan M, Aksenova V, Tentler D, Garabadgiu AV, Melino G, Barlev NA (2013). miR-16 and miR-26a target checkpoint kinases Wee1 and Chk1 in response to p53 activation by genotoxic stress. Cell death & disease.

[42] Ma H, Pederson T (2013). The nucleolus stress response is coupled to an ATR-Chk1-mediated G2 arrest. Mol Biol Cell.

[43] Johnston M, Geoffroy MC, Sobala A, Hay R, Hutvagner G (2010). HSP90 protein stabilizes unloaded argonaute complexes and microscopic P-bodies in human cells. Mol Biol Cell.

[44] Miyoshi T, Takeuchi A, Siomi H, Siomi MC (2010). A direct role for Hsp90 in pre-RISC formation in Drosophila. Nat Struct Mol Biol.

[45] Tahbaz N, Carmichael JB, Hobman TC (2001). GERp95 belongs to a family of signal-transducing proteins and requires Hsp90 activity for stability and Golgi localization. J Biol Chem.

[46] Tahbaz N, Kolb FA, Zhang H, Jaronczyk K, Filipowicz W, Hobman TC (2004). Characterization of the interactions between mammalian PAZ PIWI domain proteins and Dicer. EMBO Rep.

[47] Imai J, Maruya M, Yashiroda H, Yahara I, Tanaka K (2003). The molecular chaperone Hsp90 plays a role in the assembly and maintenance of the 26S proteasome. EMBO J.

[48] Savulescu AF, Shorer H, Kleifeld O, Cohen I, Gruber R, Glickman MH, Harel A (2011). Nuclear import of an intact preassembled proteasome particle. Mol Biol Cell.

[49] Overath T, Kuckelkorn U, Henklein P, Strehl B, Bonar D, Kloss A, Siele D, Kloetzel PM, Janek K (2012). Mapping of O-GlcNAc sites of 20 S proteasome subunits and Hsp90 by a novel biotin-cystamine tag. Mol Cell Proteomics.

[50] Davis BN, Hilyard AC, Lagna G, Hata A (2008). SMAD proteins control DROSHA-mediated microRNA maturation. Nature.

[51] Suzuki HI, Yamagata K, Sugimoto K, Iwamoto T, Kato S, Miyazono K (2009). Modulation of microRNA processing by p53. Nature.

[52] Barlev NA, Sayan BS, Candi E, Okorokov AL (2010). The microRNA and p53 families join forces against cancer. Cell death and differentiation.

[53] Zhang Y, Chang C, Gehling DJ, Hemmati-Brivanlou A, Derynck R (2001). Regulation of Smad degradation and activity by Smurf2, an E3 ubiquitin ligase. Proc Natl Acad Sci U S A.

[54] Marouco D, Garabadgiu A. V, Melino G, Barlev N.A (2013). Lysine-specific modifications of p53: a matter of life and death? Oncotarget.

[55] Wang X, Chen CF, Baker PR, Chen PL, Kaiser P, Huang L (2007). Mass spectrometric characterization of the affinity-purified human 26S proteasome complex. Biochemistry.

[56] Tsimokha AS, Mittenberg AG, Kulichkova VA, Kozhukharova IV, Gause LN, Konstantinova IM (2007). Changes in composition and activities of 26S proteasomes under the action of doxorubicin--apoptosis inductor of erythroleukemic K562 cells. Cell biology international.

[57] Kulshreshtha R, Ferracin M, Wojcik SE, Garzon R, Alder H, Agosto-Perez FJ, Davuluri R, Liu CG, Croce CM, Negrini M, Calin GA, Ivan M (2007). A microRNA signature of hypoxia. Molecular and Cellular Biology.

[58] King JY, Ferrara R, Tabibiazar R, Spin JM, Chen MM, Kuchinsky A, Vailaya A, Kincaid R, Tsalenko A, Deng DX, Connolly A, Zhang P, Yang E, Watt C, Yakhini Z, Ben-Dor A (2005). Pathway analysis of coronary atherosclerosis. Physiological genomics.

[59] Kalscheuer S, Zhang X, Zeng Y, Upadhyaya P (2008). Differential expression of microRNAs in early-stage neoplastic transformation in the lungs of F344 rats chronically treated with the tobacco carcinogen 4-(methylnitrosamino)-1-(3-pyridyl)-1-butanone. Carcinogenesis.

